# Explainable Machine Learning for Predicting Dengue Recovery Duration: Insights from Multi-Center Clinical Data

**DOI:** 10.3390/healthcare14131881

**Published:** 2026-06-27

**Authors:** Adam Khan, Asad Ali, Fazal Hanan, Muhammad Ismail Mohmand

**Affiliations:** 1 Department of Computer Science and IT, Sarhad University of Science and Information Technology, Peshawar 25000, Khyber Pakhtunkhwa, Pakistan; adam.me@suit.edu.pk; 2 Computer Engineering Department, Cyprus International University, Nicosia 99010, Cyprus; aali@ciu.edu.tr; 3 Department of Pathology, Saidu Teaching Hospital, Swat 19200, Khyber Pakhtunkhwa, Pakistan; drfhanan@gmail.com; 4 Department of Computer Engineering, Faculty of Engineering and Natural Sciences, Istanbul Atlas University, Istanbul 34408, Turkey

**Keywords:** explainable artificial intelligence, dengue recovery prediction, interpretable machine learning, clinical decision support, healthcare data analytics, patient outcome modeling

## Abstract

**Background:** Dengue fever remains a major public health challenge in endemic regions, where recovery duration varies considerably across patients due to a combination of clinical, demographic, and contextual factors. Although machine learning (ML) approaches have increasingly been applied to dengue related prediction tasks, many existing models operate as black boxes, limiting their interpretability and practical usefulness in healthcare settings. This study presents an Explainable Artificial Intelligence (XAI) based machine learning framework for analyzing dengue recovery duration using a multi-center clinical dataset collected from healthcare institutions across Khyber Pakhtunkhwa, Pakistan. **Methods:** Clinical records from 100 laboratory-confirmed dengue patients treated across multiple healthcare institutions were analyzed. The dataset included demographic, socio-economic, and clinical variables. Four machine learning models: Linear Regression, Decision Tree, Random Forest, and Neural Network, were developed and evaluated using 10-fold cross-validation. Explainability techniques, including Partial Dependence Plots (PDP), Individual Conditional Expectation (ICE), and Local Interpretable Model-Agnostic Explanations (LIME), were employed to investigate global and patient specific factors influencing recovery duration. **Results:** Among the evaluated models, Random Forest demonstrated the best overall predictive performance, achieving the lowest Root Mean Square Error (RMSE; 11.29 days) and Mean Absolute Error (MAE; 9.09 days), corresponding to a 40.4% reduction in prediction error compared with Linear Regression. Decision Tree also showed substantial improvement, reducing RMSE by 37%, whereas the Neural Network achieved a more modest improvement of 8.6%. Although all models exhibited relatively low coefficient of determination (R^2^) values (maximum R^2^ = 0.026), the explainability analyses consistently identified age and platelet count as the most influential predictors of recovery duration. Older age and lower platelet counts were generally associated with longer recovery periods, while hospital type, education level, and blood group also contributed to prediction outcomes. ICE and LIME analyses further revealed considerable patient level heterogeneity, indicating that recovery trajectories are shaped by complex interactions among clinical, demographic, and contextual factors rather than a single dominant predictor.

## 1. Introduction

Dengue is a mosquito-borne viral infection that poses a major public health threat in tropical and subtropical regions. The disease is rapidly spreading and is associated with high transmissibility, as well as significant morbidity and mortality [1]. The disease is caused by four closely related dengue virus serotypes (DENV-1–DENV-4) and is mainly transmitted by the *Aedes aegypti* mosquito, which thrives on stagnant water in densely populated urban areas [2,3,4,5]. The rates of dengue cases across the world have increased considerably over the past few decades, leading to repeated outbreaks that put a huge burden on health systems.

Beyond disease diagnosis and severity assessment, understanding recovery trajectories has important clinical and public health implications [6]. In resource-constrained healthcare systems, prolonged recovery may increase hospital occupancy, healthcare expenditures, and pressure on already limited medical resources [7]. Early identification of factors associated with delayed recovery can support patient monitoring, clinical prioritization, and more efficient allocation of healthcare resources during dengue outbreaks [8]. Consequently, recovery oriented analytics provide a complementary perspective to severity prediction by focusing on post-diagnosis patient management and healthcare planning [9].

Pakistan has faced a significant increase in dengue outbreaks in the last decade. The high rates of urbanization, poor waste disposal, and seasonal flooding have provided favorable ecological habitats for the breeding of mosquitoes and the spread of diseases [10]. Hospitals are often overwhelmed by dengue cases during outbreak periods, and timely clinical decision making and proper allocation of resources are becoming even more significant. Although most patients recover with proper treatment, some contract serious complications such as dengue hemorrhagic fever or dengue shock syndrome that might lead to organ failure or even death unless timely treatment is provided to them [11,12]. As a result, factors determining patient recovery have emerged as a central research issue in clinical and public health settings [13].

Therefore, machine learning (ML) methods have recently been applied to support medical decision making in the management of infectious diseases. Recent developments in biomedical informatics have shown that ML methods can be highly effective for uncovering complex patterns and relationships within healthcare and biological data. For instance, ref. [14] introduced a hybrid Gradient Boosting Decision Tree–Logistic Regression (GBDT-LR) framework for identifying potential microRNA (miRNA)–disease associations, demonstrating how ML can leverage heterogeneous biomedical data to uncover meaningful disease-related insights. Similarly, ref. [15], proposed the NEDD framework, which combines network representation learning with Random Forest classification to integrate diverse drug disease information and identify previously unrecognized associations. Together, these studies demonstrate the increasing value of ML in healthcare research, where it can assist in extracting actionable knowledge from large and complex datasets and support more informed decision making.

In the context of dengue, ML models have been used to predict disease severity, hospitalization risk, and outbreak dynamics [16,17,18,19]. However, many of these models function as “black boxes”, providing limited transparency regarding how predictions are generated [20,21]. In healthcare settings, such a lack of interpretability can reduce the trust of clinicians in automated systems and limit their practical adoption [22]. To address this limitation, Explainable Artificial Intelligence (XAI) techniques have emerged as an important research direction for improving model transparency and interpretability [23,24,25,26]. XAI methods allow researchers and clinicians to examine how individual features influence model predictions and identify patterns that may otherwise remain hidden within complex models. Despite growing interest in XAI, most existing studies in dengue analytics focus primarily on predicting disease severity rather than understanding recovery outcomes. Moreover, many studies rely on single-center datasets and emphasize clinical indicators alone, while demographic and socio-economic characteristics are often underexplored.

This study aims to bridge these gaps by applying explainable ML techniques to analyze dengue recovery outcomes using a multi-center clinical dataset. The dataset integrates demographic, socio-economic, and clinical attributes, allowing a comprehensive examination of the factors associated with patient recovery. Instead of focusing solely on predictive accuracy, the study emphasizes interpretability and the identification of influential features that shape recovery predictions. By combining ML models with XAI techniques such as Local Interpretable Model-Agnostic Explanations (LIME) and Partial Dependence Plots (PDP), the proposed framework provides both global and patient level explanations.

In this way, the study will produce interpretable conclusions that can help clinicians and public health decision makers understand the trends related to dengue recovery. Subsequent analysis could be used to improve clinical evaluation, better stratification of patients, and formulation of specific healthcare responses to dengue management in Pakistan and other healthcare institutions.

### 1.1. Research Gap and Objectives

Despite many approaches that have used ML methods to predict dengue related tasks, there are a number of limitations. First, recovery oriented analysis has received relatively little attention, whereas most of the existing work focuses on predictions of disease severity or mortality. Second, most studies are based on datasets that have been gathered in a medical facility, which can limit the generalizability of the results. Third, demographic and socio-economic factors, including education, income, and residential conditions, are not commonly studied together with clinical indicators in the analysis of dengue outcomes. Lastly, very few studies have used XAI methods to offer clear explanations of model predictions.

To overcome these limitations, this study investigates dengue recovery outcomes with the help of a multi-center study that combines demographic, socio-economic, and clinical factors. To this end, the main questions of our research are:

1. To apply XAI techniques to identify and rank the most important features associated with dengue patient recovery.
2. To evaluate the predictive performance and interpretability of several ML models used for recovery prediction.
3. To obtain interpretable insights that can help clinicians and public health authorities in designing targeted treatment and intervention strategies.

We emphasize that the aim is to extract interpretable insights from the data, not to achieve maximum predictive accuracy on this small dataset.

### 1.2. Contributions of the Study

The contributions of this study are summarized as follows:

- **Multi-center dengue recovery dataset analysis:** This study investigates dengue recovery using a multi-center clinical dataset collected from healthcare facilities in Khyber Pakhtunkhwa, Pakistan. The dataset integrates demographic, socio-economic, and clinical variables, enabling a context-sensitive analysis of recovery outcomes.
- **Explainability-driven machine learning framework:** An interpretable modeling framework is developed by combining multiple ML models (Linear Regression, Decision Tree, Random Forest, and Neural Network) with explainable AI techniques to analyze recovery related patterns.
- **Integration of global and local explanation methods:** The study applies complementary explainability approaches, including Partial Dependence Plots (PDP), Individual Conditional Expectation (ICE), and LIME, to capture both population-level trends and patient specific prediction behavior.
- **Interpretation of recovery related factors:** The analysis reveals how demographic, clinical, and contextual variables interact to influence dengue recovery outcomes, highlighting the importance of interpretable models for understanding heterogeneous patient recovery trajectories.

### 1.3. Research Questions

Based on these objectives, the following research questions guide this study:

- **RQ1:** Which demographic, socio-economic, and clinical features most strongly influence dengue patient recovery, and how can XAI techniques help identify these factors?
- **RQ2:** How do different ML models compare in terms of predictive performance and interpretability when predicting dengue recovery duration?
- **RQ3:** How can interpretable model explanations support clinical decision making and targeted public health interventions in dengue management?

## 2. Related Work

The growing availability of healthcare data has encouraged the application of machine learning (ML) techniques in infectious disease research. In the context of dengue, ML models have been widely used for tasks such as outbreak prediction, risk stratification, and prediction of clinical outcomes. These approaches often combine heterogeneous data sources, including demographic characteristics, laboratory indicators, and environmental variables, to improve predictive performance [27,28]. Although many studies demonstrate promising results, most focus primarily on prediction accuracy and provide limited insight into how different variables influence model decisions.

Several studies conducted in Pakistan have explored ML-based approaches for dengue analysis. For example, comparative experiments reported that Support Vector Machine (SVM) models achieved strong performance for the early diagnosis of dengue using clinical indicators [13]. Other studies investigated prognostic models to estimate hospitalization duration, mortality risk, or disease severity [5,29]. Although these investigations demonstrate the practical value of ML for dengue related healthcare analytics, they mainly emphasize predictive performance and rarely analyze how model predictions are generated or what features drive the outcomes.

As ML models become more complex, concerns about transparency and interpretability have gained increasing attention in healthcare research. Clinicians often require clear explanations of model predictions before integrating algorithmic outputs into clinical decision making processes. The Explainable Artificial Intelligence (XAI) methods aim to address this issue by providing techniques that reveal the contribution of individual features and clarify how model predictions are formed [30].

Recent studies [31,32,33,34,35] have highlighted the growing importance of explainable ML in biomedical and healthcare analytics. These investigations demonstrate that interpretable models can improve transparency, facilitate understanding of prediction mechanisms, and enhance trust in AI-assisted clinical decision-making. Such findings support the integration of explainability techniques within healthcare prediction frameworks, particularly in applications where transparency and accountability are essential.

Several model-agnostic interpretation techniques have been proposed for this purpose. Methods such as SHapley Additive exPlanations (SHAP) [36] and Local Interpretable Model-Agnostic Explanations (LIME) [37] allow researchers to analyze feature contributions at both global and local levels. These approaches have been successfully applied in various medical domains, including diagnostic imaging, disease risk prediction, and treatment outcome analysis [38,39]. By enabling transparent interpretation of complex models, XAI techniques can help improve trust and accountability in data-driven healthcare systems.

Recent work has begun to apply XAI methods to infectious disease analytics. Rahman et al. [25] utilized SHAP and LIME in combination with gradient boosting models to identify environmental and climatic drivers of dengue outbreaks in Bangladesh. Similarly, Yang et al. [40] investigated the relationship between urban land-use patterns and dengue transmission in Taiwan using explainable models. In a related study, Muriithi et al. [41] applied interpretable ML techniques to predict malaria risk in Kenya, demonstrating how transparent feature analysis can support targeted public health interventions.

Despite these developments, most of the XAI-based infectious disease studies focus on environmental or epidemiological prediction rather than patient level clinical outcomes [42,43,44]. The application of interpretable ML techniques to analyze recovery trajectories in dengue patients remains limited.

Dengue outbreaks have been common in Pakistan in recent years, which is why there has been increased research focused on epidemiological trends and clinical risk factors. Studies have been carried out in cities such as Rawalpindi and Karachi, which have indicated that there are associations between laboratory indicators, clinical manifestations, and fatal results [5,45]. Other studies have highlighted the changing patterns in serotypes and the increasing burden of disease in various parts of the country [29].

Methodological studies addressing rare or high-risk medical outcomes have also emphasized the importance of robust modeling strategies capable of handling class imbalance and heterogeneous patient populations [46,47]. However, most existing work in Pakistan continues to focus on severity classification or outbreak prediction. Relatively little attention has been paid to understanding recovery dynamics or examining how demographic and socio-economic factors interact with clinical indicators to influence patient outcomes.

Although ML has been increasingly applied to dengue analytics, relatively few studies have focused on explainability and recovery oriented outcomes. Most existing investigations prioritize disease severity prediction, outbreak forecasting, or diagnostic classification, often emphasizing predictive performance while providing limited insight into the underlying decision making process. In clinical settings, however, transparent and interpretable models are essential because healthcare professionals require explanations that can support trust, accountability, and informed decision-making. These considerations motivate the integration of explainable AI techniques within dengue recovery analysis.

Table 1 summarizes representative studies in the literature, including their geographic focus, data sources, modeling approaches, and key findings.

Even though the current literature shows that ML is useful in dengue prediction and outbreak analysis, the majority of the literature is based on severity classification or environmental forecasting. Little attention has been paid to recovery oriented modeling that incorporates demographic, socio-economic, and clinical characteristics into an interpretable framework. These limitations motivate the explainable ML approach proposed in this study.

## 3. Methodology

This paper follows an explainability-focused ML design to study dengue recovery outcomes. The methodology does not focus on large-scale optimization of features but instead focuses on the interpretability and transparent analysis of feature effects. The overall workflow includes dataset preparation, feature preprocessing, predictive modeling, and interpretation using explainable AI techniques.

### 3.1. Dataset Description

The data collection was carried out in collaboration with the Department of Pathology, Saidu Teaching Hospital, Swat, Pakistan. It contains the clinical records of the 100 confirmed dengue patients who were hospitalized in various health institutions in Khyber Pakhtunkhwa, such as Swat, Mardan, Dir, Mansehra, Chitral, Gilgit, Hunza, Skardu, and Muzaffarabad. These institutions are various healthcare settings, such as Government hospitals, privately owned hospitals, military hospitals, and clinics that are supported by NGOs.

The dataset used in this study was compiled from clinical records obtained from several healthcare institutions across Khyber Pakhtunkhwa, Pakistan. To provide greater transparency regarding the multicenter nature of the data, Table 2 presents the distribution of patients contributed by each participating hospital or region. This information helps illustrate the diverse clinical sources represented in the dataset and reflects the real-world healthcare settings from which the data were collected.

The patient cohort comprises a broad demographic range, including those between the ages of 1 and 89 years with an almost equal gender ratio (52 percent male and 48 percent female). Records with confirmed diagnoses and cases with complete demographic and clinical information were retained to ensure data reliability.

The dataset is a combination of demographic, socio-economic, and clinical features. Demographic features include age, sex, and residential area, whereas socio-economic indicators involve the level of education, financial status, and type of hospital. The clinical variables are blood group, platelet count, diabetic status, vaccination status, and the reported recovery period. Platelet counts range between 52,907 and 388,784, indicating changes in the severity of the disease.

The target variable in this study is *recovery duration*, measured as the number of days required for patient recovery following dengue infection. Recovery duration was treated as a continuous outcome variable and used directly for model development and explainability analysis. This formulation enables the investigation of factors associated with variations in recovery time rather than binary outcome classification.

### 3.2. Data Preprocessing

Prior to modeling, the dataset was reviewed and verified by medical staff to ensure consistency and accuracy. Records that contained missing, inconsistent, or duplicate information were removed during the data curation process, resulting in a fully complete dataset to be analyzed.

Feature distributions were inspected before model development to identify inconsistencies and verify data quality. Continuous variables were standardized using z-score normalization, resulting in variables with zero mean and unit variance. Feature encoding was applied according to the type of variable. Binary variables such as sex, diabetic status, and vaccination status were encoded as binary indicators. Nominal variables, such as hospital type, blood group, and residential region, were represented by one-hot encoding to avoid introducing artificial ordinal patterns. Ordinal variables, including education level and financial status, were mapped to ordered numerical scales with increasing socio-economic levels. In addition, continuous variables (age, platelets, etc., recovery time) were standardized to a zero mean and unit variance. No further feature constructions or dimensionality reduction methods were applied in order to preserve the interpretability of individual predictors.

The distribution of recovery outcomes was examined prior to model training. Since the outcome distribution was reasonably balanced across observed recovery durations, no oversampling, undersampling, or synthetic resampling techniques were applied.

### 3.3. Machine Learning Models

Four supervised ML algorithms were employed to predict dengue recovery outcomes: Linear Regression (LR) [48], Decision Tree (DT) [49], Random Forest (RF) [50], and a feed-forward Neural Network (NN) [51]. These models were selected to represent both interpretable baseline methods and more flexible nonlinear learning approaches.

The chosen algorithms encompass different degrees of complexity and interpretability. LR and DT models offer more transparent decision processes and function as baseline interpretable approaches. RF and NN models enable the investigation of more advanced nonlinear relationships among predictors. Comparing explanations from these various model types helps determine if the recovery related factors remain consistent regardless of the model structure, thus enhancing confidence in the interpretability results.

Model training and evaluation were implemented in the R environment using the caret library (R version 4.4.1) [52], which provides a unified interface for training and comparing ML models.

### 3.4. Model Training and Validation

A 10-fold cross-validation strategy was adopted to maximize utilization of the available data while reducing variance associated with a single train-test split. All models were trained using a 10-fold cross validation strategy to improve generalization and reduce the risk of overfitting [53,54,55]. The same dataset and validation procedure were applied consistently across all algorithms to maintain comparability. Given our emphasis on interpretability, we used default or minimally tuned hyperparameters for each model (as provided by the caret library) rather than extensive optimization. This avoids overfitting, given the limited sample size.

### 3.5. Model Evaluation Metrics

Since recovery duration is a continuous outcome variable, model performance was evaluated using standard regression metrics. Root Mean Squared Error (RMSE) and Mean Absolute Error (MAE) were used to quantify prediction error, while the coefficient of determination (R^2^) was used to assess the proportion of variance explained by each model. Lower RMSE and MAE values indicate better predictive performance, whereas higher R^2^ values indicate greater explanatory capability. Performance estimates were obtained using 10-fold cross-validation.

### 3.6. Explainable AI Techniques

To interpret the predictions generated by the ML models, two complementary XAI techniques were employed.

Local Interpretable Model-Agnostic Explanations (LIME) [37] was used to analyze individual predictions. LIME approximates complex models locally using simpler surrogate models, allowing the contribution of specific features to be examined for individual patient cases.

Partial Dependence Plots (PDP) [56] were used to evaluate the global influence of key features on predicted recovery probabilities. PDPs illustrate how variations in a given variable affect model predictions while averaging over the remaining features.

PDP, ICE, and LIME were selected because they provide complementary perspectives on model behavior. PDP enables examination of average feature effects across the patient population and supports identification of global recovery trends. ICE complements PDP by visualizing how feature changes affect predictions for individual patients, thereby revealing heterogeneity that population averages may hide. LIME provides localized explanations by approximating model behavior around individual instances, allowing the contribution of specific predictors to be examined for a particular patient. The combination of LIME and PDP analysis allows the study to present patient level explanations and global feature insights, which will allow a comprehensive understanding of the factors that affect dengue recovery predictions.

**Figure 1:**
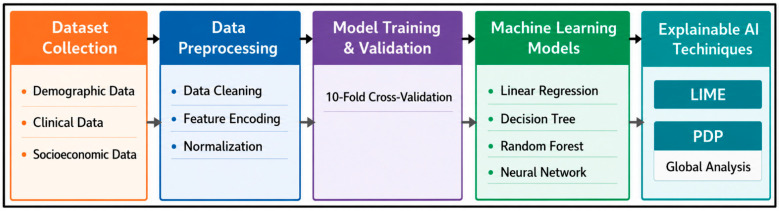
Overview of the proposed explainable ML framework for dengue recovery analysis.

Figure 1 presents the overall workflow of the proposed explainable ML framework, describing the stages of data preparation, model development, validation, and interpretation using explainable AI techniques.

## 4. Results and Discussion

This section presents the results addressing the research questions and interprets the findings obtained from the explainable ML analysis.

**RQ1:**  *WHICH DEMOGRAPHIC, SOCIO-ECONOMIC, AND CLINICAL FEATURES MOST STRONGLY INFLUENCE DENGUE PATIENT RECOVERY, AND HOW CAN XAI TECHNIQUES HELP IDENTIFY THESE FACTORS?*

In order to determine the crucial factors that affect the recovery from dengue, we evaluate the model explanations through global and local explainable AI methods. Partial Dependence Plots (PDP) are used to analyze global trends, and Individual Conditional expectation (ICE) curves and LIMEs are used to analyze at the patient level.

### 4.1. Global Feature Effects

**Figure 2:**
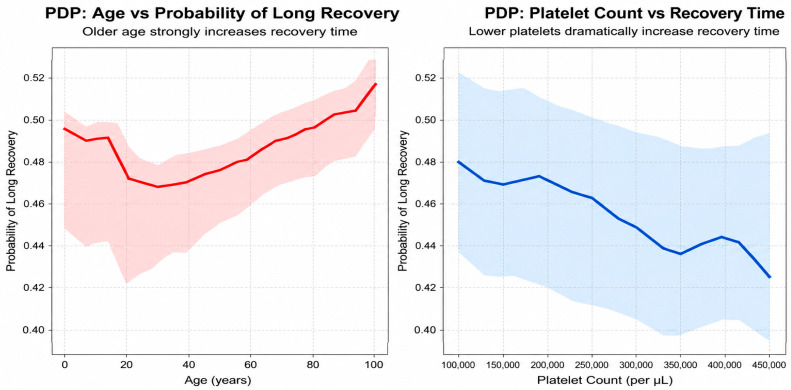
Partial dependence plots (PDPs) showing the influence of age and platelet count on the predicted probability of prolonged dengue recovery. The solid lines represent the average partial dependence, while the shaded areas indicate the associated uncertainty.

Figure 2 presents the partial dependence plots (PDPs) for the two most clinically relevant numerical variables: age and platelet count. The PDP for age demonstrates a gradual increase in the predicted probability of prolonged recovery with advancing age, particularly beyond approximately 55 years. This trend indicates that older patients are at an increased risk of delayed recovery, consistent with established clinical evidence that aging is associated with reduced physiological resilience and slower recovery from infectious diseases.

In contrast, platelet count exhibits an inverse relationship with the predicted probability of prolonged recovery. Lower platelet counts are associated with a higher likelihood of delayed recovery, whereas higher platelet counts correspond to a reduced probability of prolonged recovery. This finding is consistent with the clinical significance of thrombocytopenia as a well-established indicator of dengue severity, reinforcing the importance of platelet count as a key predictor of recovery duration.

### 4.2. Insights from XAI on Recovery Determinants

Age appeared to have an important influence on recovery outcomes. This may be because older individuals generally have a lower capacity to recover from infectious illnesses compared to younger patients. In many cases, advancing age is also accompanied by other health conditions that can complicate recovery and prolong the healing process. Platelet count was another key factor identified in the analysis, which aligns with its well-recognized role in dengue management. Patients with lower platelet levels often experience more severe manifestations of the disease and may require closer clinical monitoring. The impact of hospital type could be associated with differences in available facilities, diagnostic services, healthcare resources, and treatment practices. Education level may also contribute indirectly to recovery outcomes, as individuals with higher educational attainment may be more likely to recognize symptoms early, seek timely medical care, and follow medical advice more consistently.

Although education level was identified as an important predictor, this finding should not be viewed as evidence of a direct effect on dengue recovery. Rather, education may reflect a range of underlying factors, such as health awareness, health literacy, socio-economic status, access to healthcare services, and the likelihood of seeking timely medical attention. These factors can influence how patients recognize symptoms, follow treatment recommendations, and engage with healthcare providers. Therefore, the relationship between education and recovery outcomes should be interpreted with caution, as other unmeasured factors may also contribute to the observed association.

### 4.3. Influence of Categorical Variables

**Figure 3:**
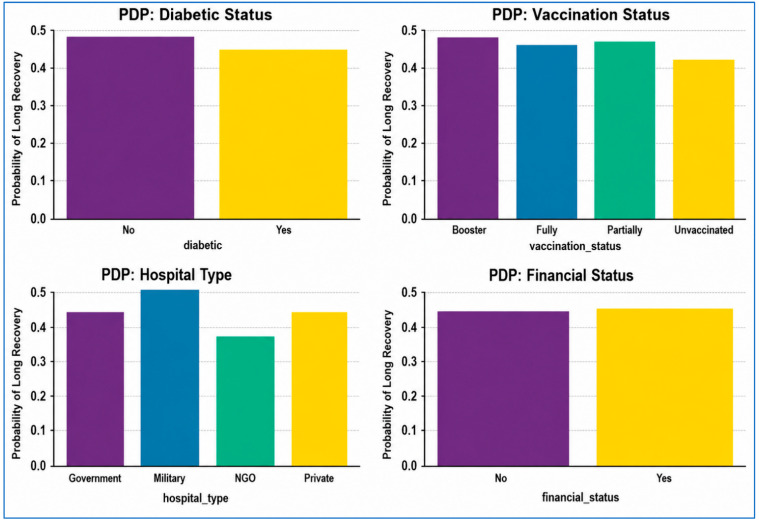
Partial dependence for categorical features.

The global effects of categorical features are shown in Figure 3. Among these variables, hospital type demonstrates the most noticeable variation in predicted recovery probabilities. Differences between hospital categories can reflect variations in healthcare infrastructure, treatment capacity, or case severity across institutions.

Alternatively, the global impact of vaccination status, diabetic status, and financial status in the model is relatively small. Such factors individually cause small differences in recovery predictions, indicating that their effects are likely to be conditional on the other variables, rather than dominating predictors.

### 4.4. Feature Interactions

**Figure 4:**
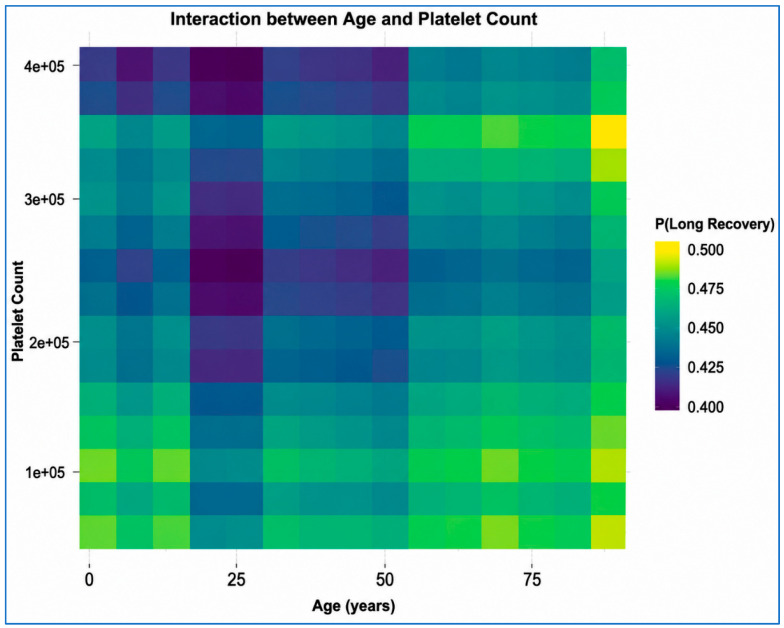
2D partial dependence plot showing interaction between age and platelet count.

To further explore feature relationships, the two dimensional PDP in Figure 4 illustrates the interaction between age and platelet count. The highest predicted probability of prolonged recovery occurs when both risk factors are present simultaneously, specifically, low platelet counts combined with advanced age. Conversely, younger individuals with higher platelet counts fall into the lowest-risk region of the prediction space. This interaction indicates that platelet count acts as a dominant driver of recovery risk, while age amplifies its effect.

### 4.5. Individual-Level Variability

**Figure 5:**
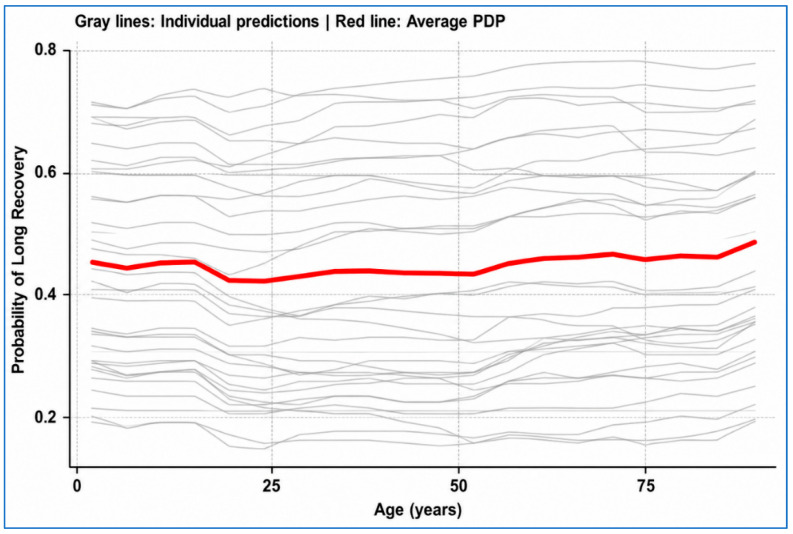
ICE (Individual Conditional Expectation) plot illustrating the individual-level effects of age on the predicted probability of long recovery. Gray lines represent the ICE curves for individual observations, while the red line represents the average Partial Dependence Plot (PDP), summarizing the overall marginal effect of age.

While PDPs provide global trends, ICE plots reveal how individual predictions respond to feature changes. Figure 5 shows the ICE curves for age. Although the average trend indicates increasing recovery time with age, the individual trajectories vary considerably, particularly among younger patients. This variability suggests that age alone does not uniformly determine recovery outcomes but interacts with other clinical and demographic factors.

The observed influence of hospital type may partially reflect differences in healthcare infrastructure, resource availability, clinical workflows, and patient management practices across participating institutions. Variations in diagnostic facilities, staffing levels, and patient-management procedures may contribute to differences in recovery patterns. Since detailed treatment-protocol information was unavailable within the dataset, these findings should be interpreted as contextual associations rather than direct evidence of treatment effectiveness.

#### Predictive Performance of Recovery Duration Models

Table 3 presents the predictive performance of the evaluated regression models using 10-fold cross-validation. Because recovery duration is a continuous outcome variable, regression-based metrics were used to assess model performance. Among the tested models, the Random Forest achieved the best overall performance, producing the lowest RMSE (11.29 days) and MAE (9.09 days), corresponding to a 40.4% reduction in prediction error compared with the baseline Linear Regression model. The Decision Tree also demonstrated substantial improvement, reducing RMSE by 37%, whereas the Neural Network achieved only a modest improvement of 8.6%. Although Random Forest outperformed the other models in terms of prediction accuracy, all models yielded relatively low *R*^2^ values, with the highest *R*^2^ being 0.026 for Linear Regression. These findings suggest that recovery duration is influenced by multiple clinical, biological, and patient specific factors that may not be fully captured in the available dataset. Therefore, the predictive models should be viewed primarily as tools for identifying patterns and supporting clinical interpretation rather than as highly accurate predicting systems. Overall, the Random Forest model was selected for subsequent analyses because it provided the most reliable balance of prediction accuracy and robustness among the evaluated approaches.

### 4.6. Local Feature Importance

**Figure 6:**
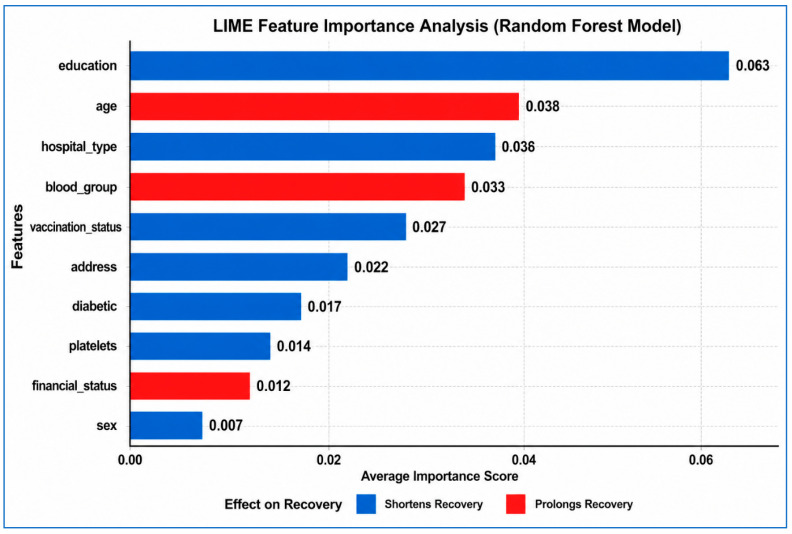
LIME-based feature importance for individual predictions.

To understand how specific features influence individual predictions, we analyze LIMEs shown in Figure 6.

Age consistently emerges as the strongest contributor to prolonged recovery, while education and certain socio-economic indicators often contribute toward shorter recovery durations. Hospital type and blood group also influence individual predictions, although their effects are less consistent across patients.

These results demonstrate that recovery predictions are shaped by combinations of demographic, clinical, and contextual variables rather than by a single dominant factor.

### 4.7. Complementary Statistical Insights

**Figure 7:**
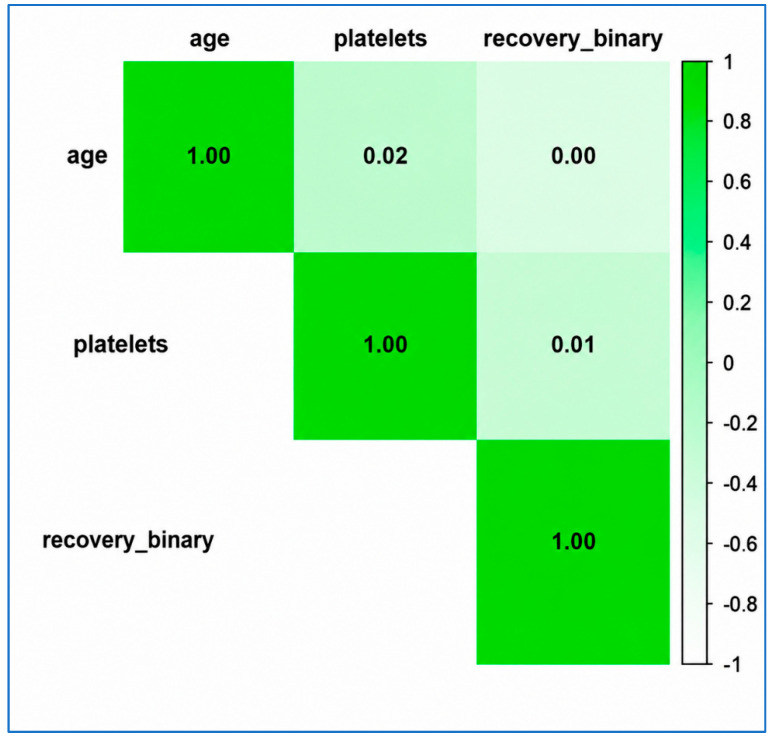
Correlation heatmap of numerical features.

To examine linear relationships between numerical variables, Figure 7 presents a correlation heatmap for age, platelet count, and recovery outcome. All pairwise correlations are weak, indicating that recovery outcomes cannot be explained through simple linear relationships between individual variables. Instead, the results support the earlier findings that recovery patterns are driven by non-linear interactions captured by the ML models.

**Figure 8:**
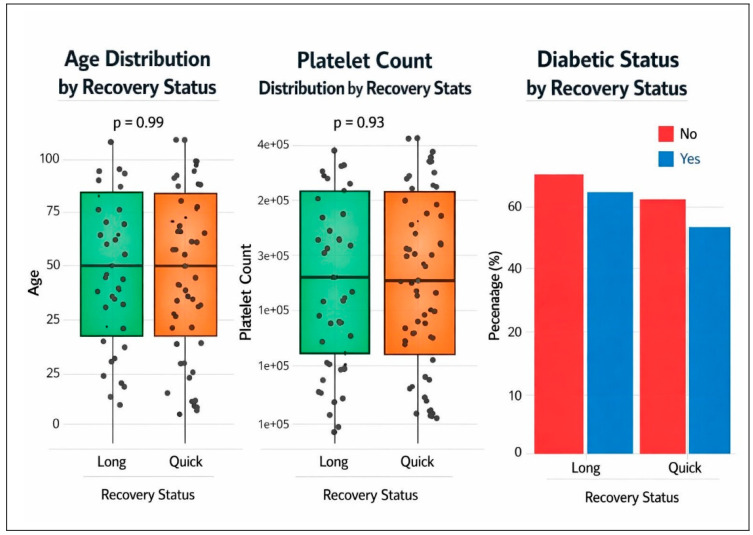
Comparison of age, platelet count, and diabetic status by recovery status—boxplots and bar charts compare age, platelet count, and diabetic status between long and quick recovery groups, showing no significant differences (*p* > 0.9).

Finally, Figure 8 compares feature distributions across recovery groups. The substantial overlap between groups further indicates that no single demographic or clinical factor alone separates quick and prolonged recovery cases. This observation reinforces the importance of explainable ML approaches that capture multivariate and non-linear relationships among features.

The considerable variation observed across the ICE curves and LIMEs indicates that dengue recovery trajectories can differ substantially among patients. While the model identifies broad recovery patterns, individual outcomes are shaped by distinct combinations of demographic, socio-economic, and clinical characteristics. These findings reflect the multifactorial nature of dengue progression and underscore the importance of incorporating patient specific information when assessing recovery potential. Such observations align with the principles of personalized medicine, where risk evaluation and clinical management are adapted to the unique circumstances of each patient. In this regard, explainable ML offers added clinical value by providing transparent insights into how different factors contribute to recovery predictions at the individual level.

Overall, the explainability analysis demonstrates that dengue recovery is influenced by a network of interacting predictors rather than a single dominant factor. Age and platelet count emerged as the most consistently influential variables across the models, whereas socio-economic and contextual attributes contributed additional explanatory power. By combining global and local interpretability techniques, the proposed framework enhances transparency in predictive modeling and provides a comprehensive understanding of the mechanisms underlying recovery outcomes. This integrated approach can support clinicians in interpreting model predictions with greater confidence while maintaining a clear link between predictive performance and clinical relevance.

**RQ2:**  *HOW DO DIFFERENT ML MODELS COMPARE IN TERMS OF PREDICTIVE PERFORMANCE AND INTERPRETABILITY WHEN PREDICTING DENGUE RECOVERY DURATION?*

**Figure 9:**
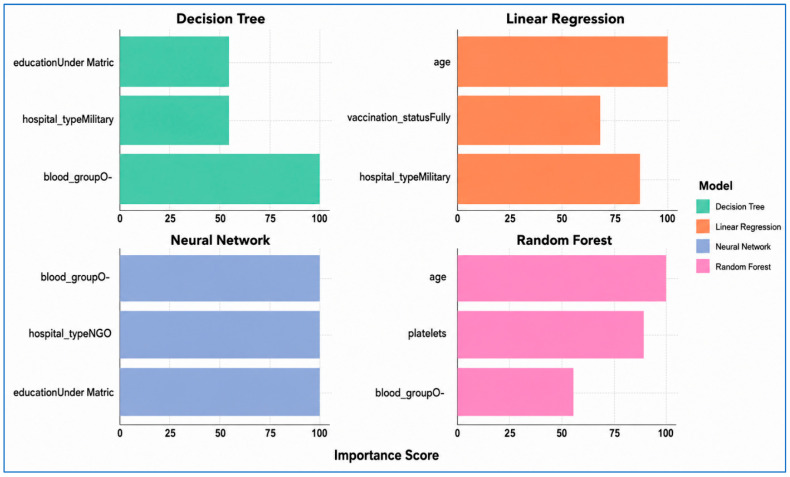
Comparative feature importance across machine learning models. Top-ranked features identified by Decision Tree, Linear Regression, Neural Network, and Random Forest models for recovery time prediction.

To compare how different ML models interpret recovery patterns, we analyze the feature importance rankings generated by each model. Figure 9 summarizes the most influential variables identified by the Decision Tree, Linear Regression, Neural Network, and Random Forest models.

Although models differ in terms of their learning mechanisms, there are a few common signals that are observed in most of them. Age, type of hospital, level of education, and blood group are recurrently effective predictors, which means that these factors play an important role in the prediction of recovery, irrespective of the model architecture.

Some model specific differences are also noticed. For instance, the Decision Tree focuses on education and hospital type as important decision splitting, which is a way to emphasize the hierarchical nature of the partitioning of the prediction process. However, Linear Regression gives more importance to age and vaccination status, which is in line with its linear formulation of risk contributions. In addition, the Neural Network highlights patterns involving the blood group and the hospital type, which may indicate that it captures relationships between variables that are more complex. Meanwhile, the Random Forest shows that age and platelet count are the primary predictors, which is close to the explanation results in the global explanation presented earlier.

Overall, the comparison shows that although individual models emphasize different predictors, several variables consistently influence the predictions between algorithms. This consistency between models strengthens the confidence that the identified factors reflect meaningful recovery related patterns rather than artifacts of a single modeling approach.

**Figure 10:**
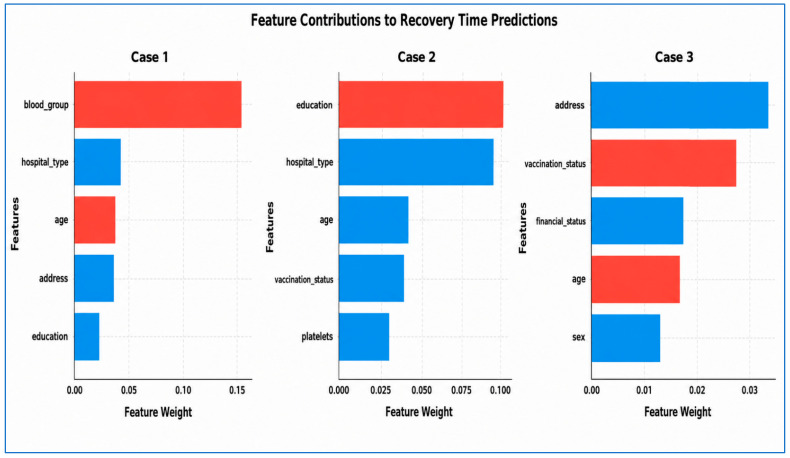
LIMEs for individual patient cases. Top contributing features and their corresponding weights for three representative patient cases, illustrating how the proposed model predicts recovery time on a patient specific basis.

To further examine interpretability at the patient level, Figure 10 presents LIMEs for three representative cases. These examples demonstrate how different features contribute to individual recovery predictions.

In all cases, the model explanations indicate that recovery outcomes are based on combinations of clinical, demographic, and contextual variables. For example, in some cases, biological factors, such as blood group or age, increase the likelihood of a longer recovery, while socio-economic or healthcare-related factors, such as education level or type of hospital, change predictions to a more rapid recovery.

These case-level explanations highlight an important characteristic of medical prediction models: the outcome for each patient depends on the interaction of multiple factors rather than a single dominant variable. By providing transparent explanations of these interactions, LIME enables clinicians to understand the reasoning behind individual predictions.

**Figure 11:**
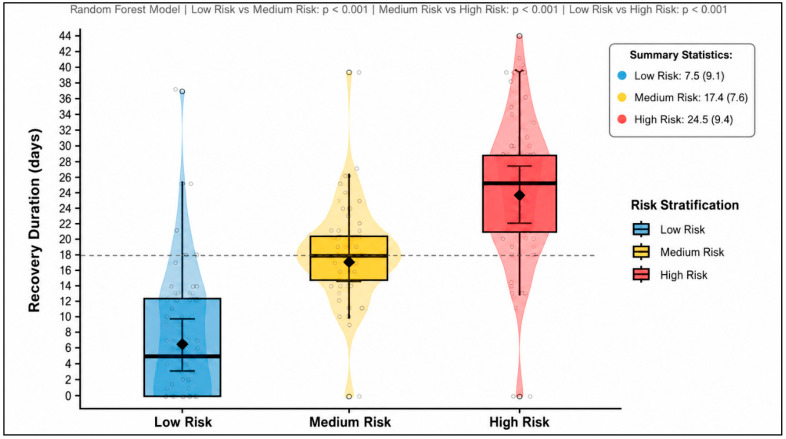
Violin plots illustrate the distribution of predicted recovery durations (days) for the Low-, Medium-, and High-Risk groups. The gray circles represent individual patient observations (with slight horizontal jitter to reduce overlap). Embedded box plots show the median, interquartile range (IQR), and 1.5 × IQR whiskers, while black diamonds indicate the mean recovery duration. The dashed horizontal line marks the overall reference level of 18 days. Summary statistics are presented as mean (SD): Low Risk = 7.5 (9.1), Medium Risk = 17.4 (7.6), and High Risk = 24.5 (9.4). Pairwise comparisons between all risk categories were statistically significant (*p* < 0.001), indicating progressively longer recovery durations with increasing risk severity.

Finally, Figure 11 shows a clear relationship between risk level and predicted recovery duration. Patients in the Low-Risk group were expected to recover the fastest, with an average recovery time of about 7.5 days. In contrast, Medium-Risk patients required approximately 17.4 days, while High-Risk patients had the longest recovery period, averaging 24.5 days. The results indicate that recovery duration increases steadily as risk severity increases. The distributions also show that High-Risk patients experienced greater variation in recovery times, suggesting that their outcomes are less predictable compared to those in the Low-Risk group. Furthermore, the statistically significant differences (*p* < 0.001) between all risk categories confirm that the model effectively distinguishes patients according to their expected recovery duration. Overall, these findings demonstrate that the Random Forest model can successfully stratify patients into meaningful risk groups and provide valuable insights for clinical decision making and recovery planning.

**RQ3:**  *HOW CAN INTERPRETABLE MODEL EXPLANATIONS SUPPORT CLINICAL DECISION-MAKING AND TARGETED PUBLIC HEALTH INTERVENTIONS IN DENGUE MANAGEMENT?*

**Figure 12:**
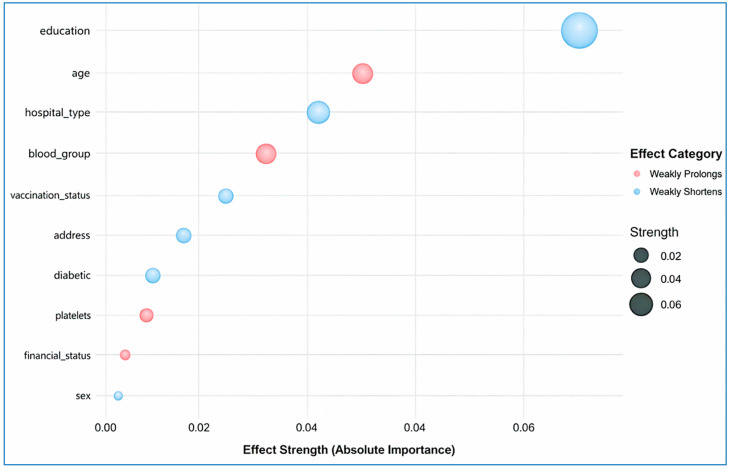
Classification by impact strength and direction—The plot shows the relative influence of predictor variables on recovery outcomes, with color indicating direction (blue = shortens, red = prolongs) and marker size representing effect strength.

To evaluate the practical implications of the explainability analysis, Figure 12 summarizes the directional influence of key features on predicted recovery time. The visualization highlights whether each variable tends to increase or decrease the likelihood of prolonged recovery.

A small set of variables, most notably age and certain blood groups, tends to shift predictions toward longer recovery durations. These effects are consistent with earlier analyses and reflect biological or physiological risk factors that may influence disease progression.

In contrast, several socio-demographic and contextual variables show a mild protective influence by shifting predictions toward shorter recovery times. Education level, hospital type, vaccination status, and residential context all contribute to this trend, suggesting that access to healthcare resources, awareness, and preventive measures may support faster recovery in some patients.

To facilitate interpretation of the explainability results, Table 4 summarizes the potential clinical and contextual significance of the most influential predictors identified across multiple ML models.

**Figure 13:**
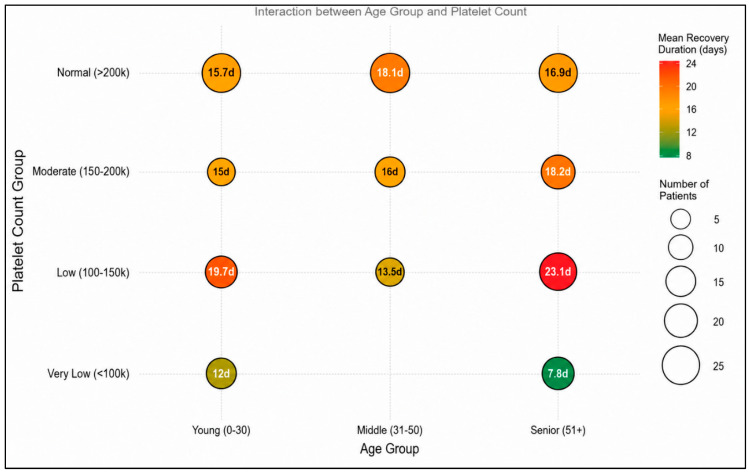
Interaction effects of age and platelet count on long-recovery proportions—The figure shows how age and platelet count interact to influence extended recovery outcomes, with color indicating recovery proportion and marker size reflecting patient sample.

Figure 13 illustrates how age group and platelet count interact to influence recovery duration. Overall, recovery time varies across different combinations of age and platelet levels, indicating that neither factor acts alone. The longest average recovery duration was observed among senior patients with low platelet counts (23.1 days), suggesting that older individuals may require more time to recover when platelet levels are reduced. In contrast, some groups, such as seniors with very low platelet counts, showed shorter recovery durations, highlighting the complexity and variability of patient outcomes. Patients with normal or moderate platelet counts generally exhibited recovery times between 15 and 18 days across all age groups. These findings suggest that recovery duration is influenced by the combined effects of age and platelet count, but the relationship is not entirely consistent. Overall, the figure supports the idea that dengue recovery is driven by multiple interacting clinical factors rather than a single predictor.

Together, these results demonstrate how explainable ML can support more transparent clinical decision making. By revealing how demographic, clinical, and contextual factors interact within predictive models, the proposed framework provides interpretable insights that can help clinicians in identifying patients at higher risk of delayed recovery. Moreover, the findings highlight how contextual variables such as education and healthcare access may influence recovery patterns, offering potential guidance for targeted public health interventions in Pakistan.

Overall, the findings suggest that an interplay of clinical, demographic, and contextual determinants governs dengue recovery. The application of explainable ML methods demonstrates that, although biological parameters such as age and platelet count are important contributors to the prediction of recovery, socio-demographic variables, including educational attainment and access to healthcare services, also substantially influence recovery trajectories. These results underscore the utility of interpretable modeling approaches for elucidating complex recovery dynamics and for informing evidence-based clinical management and public health policy.

**Figure 14:**
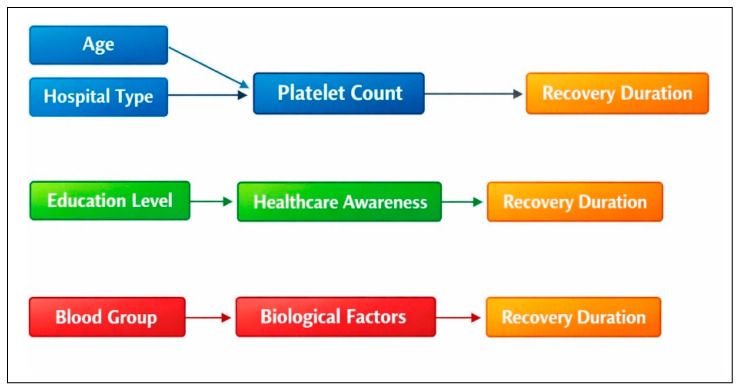
Conceptual pathway linking identified predictors to recovery outcomes.

Figure 14 presents a conceptual interpretation of how demographic, clinical, and contextual variables may collectively influence recovery outcomes. The figure is intended to support the interpretation of the explainability results rather than imply causal relationships.

## 5. Limitations of the Study

Despite providing useful insights, this study has several limitations. First, the analysis is based on a multi-center dataset collected from healthcare facilities in Khyber Pakhtunkhwa (KP), Pakistan. Although this dataset reflects real clinical practice in the region, the findings may not be fully generalized to other geographic areas with different healthcare systems or dengue transmission dynamics. In addition, information regarding detailed treatment protocols, medication regimens, and hospital specific management strategies was not available in the dataset. Consequently, potential differences in clinical practice across participating institutions could not be explicitly evaluated.

One limitation of this study is the relatively small number of patients included in the analysis. A limited sample can affect the stability of model estimates and may reduce the extent to which the findings can be generalized to other populations. For this reason, the results should be considered exploratory and interpreted with appropriate caution. Rather than providing definitive predictive conclusions, the study offers preliminary insights into factors that may be associated with dengue recovery. Future research involving larger and more diverse patient cohorts will be important for validating these findings and determining their applicability across different healthcare settings.

Second, while the dataset incorporates demographic, socio-economic, and clinical variables, other potentially relevant factors, such as treatment protocols, disease severity indicators, and follow-up practices, were not available and can influence recovery outcomes.

Third, the retrospective nature of the data may introduce limitations related to completeness of the data and the consistency of the reporting, which could affect model learning and interpretation.

We also note that more advanced ensemble methods (e.g., XGBoost, LightGBM) and model-agnostic explainers (e.g., SHAP) were not included in this study and represent opportunities for future research.

In addition, while PDP and ICE plots are useful tools for understanding how individual features influence model predictions, their interpretations should be approached with caution when predictors are strongly correlated. In such cases, the averaging process used to generate PDPs may produce combinations of feature values that are unlikely or even impossible in real clinical scenarios. As a result, the patterns observed in PDP and ICE analyses may not always reflect realistic patient profiles. Therefore, these visualizations are best viewed as exploratory tools that complement, rather than replace, clinical expertise and other explainability techniques. Their findings should be interpreted alongside domain knowledge and supporting evidence rather than being considered direct indicators of causal relationships.

Although LIME provides useful insights into the factors influencing individual predictions, its explanations are based on local approximations of model behavior generated through perturbations of the input data. As a result, the explanations may vary to some extent across different runs or sampling settings. For this reason, LIME outputs should be viewed as approximate interpretations of how the model behaves within a particular region of the feature space rather than as definitive evidence of causal relationships. Future studies could further examine the stability and consistency of these explanations by conducting repeated analyses and comparing LIME with other explainable AI approaches.

## 6. Conclusions

This study explored dengue recovery dynamics using an explainable ML framework applied to a multi-center clinical dataset collected from healthcare facilities in Pakistan. The objective was to go beyond purely predictive models and provide transparent insights into how demographic, clinical, and contextual variables influence patient recovery outcomes. The results demonstrate that dengue recovery is shaped by multiple interacting factors rather than by a single dominant predictor. Across different models and explainability techniques, variables such as age, platelet count, hospital type, education level, and blood group repeatedly emerged as influential contributors to recovery predictions. Although biological indicators remain important, the analysis also highlights the role of contextual and socio-demographic conditions in shaping recovery trajectories.

By combining global and local explanation methods, including PDP, ICE, and LIME, the proposed framework provides interpretable insights into both population-level trends and patient specific variations. These explanations reveal substantial heterogeneity in recovery patterns, indicating that individual outcomes depend on complex combinations of risk and protective factors.

This study offers an exploratory examination of dengue recovery outcomes using a multicenter clinical dataset collected in Pakistan and interpreted through explainable ML techniques. The findings indicate that recovery is likely influenced by a combination of clinical, demographic, and contextual factors, reflecting the complex nature of disease progression and patient response. However, the observed relationships should be interpreted as associations rather than evidence of causation. Given the relatively limited sample size, the results are best considered as preliminary insights that can help generate new hypotheses and inform future research. Despite these limitations, the study demonstrates how interpretable machine learning approaches can contribute to a better understanding of recovery patterns and may serve as a useful foundation for future work on dengue outcome prediction and clinical decision support.

The findings presented in this study should be regarded as exploratory observations derived from a clinically relevant multicenter dataset. Although the explainability analyses revealed several interesting patterns related to recovery outcomes, these results are intended to aid interpretation and generate new research questions rather than establish firm causal relationships. The identified patterns may help guide future investigations, but additional evidence from larger studies will be needed before broader conclusions can be drawn.

To the best of our knowledge, this study represents one of the first analyses based on the explainability of dengue recovery using a multi-center clinical dataset from Pakistan.

## 7. Future Work

Future research can expand this work by incorporating larger and more diverse patient datasets to capture broader recovery dynamics across different regions and healthcare settings. Including additional clinical indicators, such as disease severity markers, treatment details, and longitudinal laboratory measurements, may further improve the understanding of recovery trajectories.

In addition, temporal modeling approaches could better represent the evolving progression of dengue infection and recovery over time. Exploring advanced ML frameworks and risk stratification methods may further enhance predictive performance, facilitate the identification of clinically significant patient subgroups, and support more targeted treatment strategies.

Another promising direction for future research is the integration of the proposed prediction and explainability framework into real-world clinical decision-support systems. This could involve incorporating the models into hospital information systems or point-of-care applications, while ensuring that predictions and explanations can be generated efficiently enough to support timely clinical decision-making. Particular attention should also be given to deployment in health care settings with limited computational resources, where model efficiency and scalability are especially important. In addition, future studies should explore the development of intuitive and clinician-friendly interfaces for presenting PDP, ICE, and LIMEs. Evaluations involving healthcare professionals would help determine whether these explanations are easy to understand, clinically relevant, and useful for decision-making. Such investigations could also provide valuable insights into user trust, cognitive workload, interface design preferences, and other factors that may influence the successful adoption of explainable AI technologies in everyday clinical practice.

Finally, external validation using datasets collected from different hospitals and geographic regions will be essential to assess the generalizability, robustness, and real-world applicability of the proposed explainable ML framework.

## Figures and Tables

**Table 1:** Summary of key characteristics of prior dengue prediction studies.

#	Authors	Country/ Region	Dataset Type & Size		Outcome /Target	Methods	Key Findings
1	Aziz & Aziz (2021) [27]	Pakistan	Time-series data		Case forecasting	ML models	ML is effective for forecasting; it lacks interpretability.
2	Saeed et al. (2024) [28]	Pakistan	Monthly cases/deaths		Classification	Regression, ML	Demonstrated ML usefulness; no explainability/XAI applied.
3	Hanan et al. (2022) [5]	Pakistan (Rawalpindi)	Hospital records	patient	Hospital stay & outcomes	Statistical/ML	Identified prognostic clinical features.
4	Riaz et al. (2024) [29]	Pakistan	Multi-center dataset	clinical	Risk factors for DHF	Retrospective analysis	Highlighted key clinical factors contributing to DHF severity.
5	Usmani et al. (2025) [45]	Pakistan (Karachi)	66 patient records		Severe dengue progression	Clinical analysis	AST and platelet levels were found to be important predictors of severity.
6	Rahman (2025) et al. [25]	Bangladesh	2000–2023 epidemiological & climatic data		Outbreak prediction	XGBoost + SHAP/LIME	Identified major eco-climatic factors influencing dengue outbreaks.
7	Yang et al. (2023) [40]	Taiwan	Land-use & environmental datasets		Spatial distribution	XGBoost + SHAP	Revealed strong environmental influence on dengue spread.
8	Muriithi (2025) et al. [41]	Kenya	Malaria risk dataset		Risk prediction	RF, XGBoost + SHAP	Validated XAI models for infectious disease risk prediction.
9	Hayat et al. (2024) [13]	Pakistan	Clinical dataset	patient	Diagnosis/classification	ANN	High classification accuracy; lacks model interpretability.

**Table 2:** Distribution of patients across participating regions.

Hospital/Region	Number of Patients	Percentage (%)
Gilgit	16	16.0
Hunza	15	15.0
Mansehra	15	15.0
Skardu	9	9.0
Mardan	9	9.0
Chitral	8	8.0
Swat	8	8.0
Dir	7	7.0
Muzaffarabad	7	7.0
Abbottabad	6	6.0
**Total**	**100**	**100.0**

**Table 3:** Performance comparison of machine learning models for recovery time prediction.

Model	RMSE	MAE	R2	RMSE_Improvement
Linear Regression	18.94	15.68	0.026	0
Decision Tree	11.93	10.08	0.004	37
Random Forest	11.29	9.09	0.012	40.4
Neural Network	17.31	13.87	0.024	8.6

**Table 4:** Clinical interpretation of key predictors identified by explainable machine learning models.

Predictor	Observed Effect on Recovery	Potential Clinical Interpretation
Age	Higher age associated with prolonged recovery	Reduced physiological resilience, weakened immune responsiveness, and increased susceptibility to severe disease outcomes.
Platelet Count	Lower platelet count associated with prolonged recovery	Potential marker of disease severity, systemic inflammation, and increased risk of clinical complications.
Hospital Type	Variable effect across institutions	May reflect differences in healthcare resources, treatment protocols, patient management strategies, and accessibility of specialized care.
Education Level	Context-dependent effect	Can serve as a proxy for health literacy, awareness of preventive measures, healthcare-seeking behavior, and treatment adherence.
Blood Group	Variable influence	Suggests a possible biological association with disease progression and recovery, warranting further clinical investigation.

## Data Availability

The datasets used and/or analyzed during the current study are publicly available on Kaggle at: https://www.kaggle.com/datasets/adamsuit123/dengue-data. (accessed on 16 June 2026).
